# Effects of sigh during pressure control and pressure support ventilation in pulmonary and extrapulmonary mild acute lung injury

**DOI:** 10.1186/s13054-014-0474-4

**Published:** 2014-08-12

**Authors:** Lillian Moraes, Cíntia Lourenco Santos, Raquel Souza Santos, Fernanda Ferreira Cruz, Felipe Saddy, Marcelo Marcos Morales, Vera Luiza Capelozzi, Pedro Leme Silva, Marcelo Gama de Abreu, Cristiane Sousa Nascimento Baez Garcia, Paolo Pelosi, Patricia Rieken Macedo Rocco

**Affiliations:** Laboratory of Pulmonary Investigation, Carlos Chagas Filho Biophysics Institute, Federal University of Rio de Janeiro, Centro de Ciências da Saúde, Avenida Carlos Chagas Filho, s/n, Bloco G-014, Ilha do Fundão, 21941-902 Rio de Janeiro, RJ Brazil; Laboratory of Experimental Surgery, Faculty of Medicine, Federal University of Rio de Janeiro, Avenida Pedro Calmon, 550, Rio de Janeiro, RJ 21941-901 Brazil; Hospital Pró-Cardíaco, Rua General Polidoro, 192, Rio de Janeiro, RJ 22280-003 Brazil; Hospital Copa D’Or, Rua Figueiredo Magalhães, Rio de Janeiro, RJ 22031-011 Brazil; Laboratory of Cellular and Molecular Physiology, Carlos Chagas Filho Biophysics Institute, Federal University of Rio de Janeiro, Avenida Pedro Calmon, 550, Rio de Janeiro, RJ 21941-901 Brazil; Department of Pathology, School of Medicine, University of São Paulo, Avenida Prof. Almeida Prado, 1280, São Paulo, SP 05508-070 Brazil; Pulmonary Engineering Group, Department of Anesthesiology and Intensive Care Therapy, University Hospital Carl Gustav Carus, Dresden University of Technology, Mommsenstraße 11, 01069 Dresden, Germany; Rio de Janeiro Federal Institute of Education, Science and Technology, Rua Prof. Carlos Wenceslau, 343, Rio de Janeiro, RJ 25715-000 Brazil; Department of Surgical Sciences and Integrated Diagnostics, University of Genoa, IRCCS AOU San Martino-IST, Largo Rosanna Benzi 10, I-16132 Genoa, Italy

## Abstract

**Introduction:**

Sigh improves oxygenation and lung mechanics during pressure control ventilation (PCV) and pressure support ventilation (PSV) in patients with acute respiratory distress syndrome. However, so far, no study has evaluated the biological impact of sigh during PCV or PSV on the lung and distal organs in experimental pulmonary (p) and extrapulmonary (exp) mild acute lung injury (ALI).

**Methods:**

In 48 Wistar rats, ALI was induced by *Escherichia coli* lipopolysaccharide either intratracheally (ALIp) or intraperitoneally (ALIexp). After 24 hours, animals were anesthetized and mechanically ventilated with PCV or PSV with a tidal volume of 6 mL/kg, FiO_2_ = 0.4, and PEEP = 5 cmH_2_O for 1 hour. Both ventilator strategies were then randomly assigned to receive periodic sighs (10 sighs/hour, Sigh) or not (non-Sigh, NS). Ventilatory and mechanical parameters, arterial blood gases, lung histology, interleukin (IL)-1β, IL-6, caspase-3, and type III procollagen (PCIII) mRNA expression in lung tissue, and number of apoptotic cells in lung, liver, and kidney specimens were analyzed.

**Results:**

In both ALI etiologies: (1) PCV-Sigh and PSV-Sigh reduced transpulmonary pressure, and (2) PSV-Sigh reduced the respiratory drive compared to PSV-NS. In ALIp: (1) PCV-Sigh and PSV-Sigh decreased alveolar collapse as well as IL-1β, IL-6, caspase-3, and PCIII expressions in lung tissue, (2) PCV-Sigh increased alveolar-capillary membrane and endothelial cell damage, and (3) abnormal myofibril with Z-disk edema was greater in PCV-NS than PSV-NS. In ALIexp: (1) PSV-Sigh reduced alveolar collapse, but led to damage to alveolar-capillary membrane, as well as type II epithelial and endothelial cells, (2) PCV-Sigh and PSV-Sigh increased IL-1β, IL-6, caspase-3, and PCIII expressions, and (3) PCV-Sigh increased the number of apoptotic cells in the lung compared to PCV-NS.

**Conclusions:**

In these models of mild ALIp and ALIexp, sigh reduced alveolar collapse and transpulmonary pressures during both PCV and PSV; however, improved lung protection only during PSV in ALIp.

**Electronic supplementary material:**

The online version of this article (doi:10.1186/s13054-014-0474-4) contains supplementary material, which is available to authorized users.

## Introduction

Lung-protective mechanical ventilation with low tidal volume (V_T_) and positive end-expiratory pressure (PEEP) has been recommended to improve outcome in patients with acute respiratory distress syndrome (ARDS) [1]. However, low V_T_ may yield a progressive derecruitment with atelectasis, leading to deterioration in respiratory function, cyclical opening and closing of peripheral airways and alveoli, and ventilator-induced lung injury (VILI) [2]. Recruitment maneuvers (RMs) have been proposed to open collapsed lung tissue and improve oxygenation in ARDS patients [3]. Sigh, a cyclically delivered RM, effectively counteracts the tendency of lung collapse associated with low V_T_, thus improving respiratory function in ARDS patients both in controlled ventilation [4,5] and in pressure support ventilation (PSV) [6]. However, sigh increases stress/strain, possibly leading to higher biological impact. In PSV, transpulmonary pressure, ventilation, and perfusion are more homogeneously distributed [7], favoring sigh to improve respiratory function and attenuate VILI as compared with pressure-controlled ventilation (PCV). Furthermore, lung recruitability differs according to the etiology of acute lung injury (ALI). Whereas alveolar edema and tissue consolidation predominate in pulmonary ALI (ALIp), extrapulmonary ALI (ALIexp) is associated with potentially recruitable alveolar collapse [8-10]. Based on the foregoing, we hypothesized that in ALIexp, but not in ALIp, sigh combined with PSV would be more effective at opening atelectatic lung regions, thus improving lung morphofunction, with less VILI, than sigh combined with PCV.

In the present study, we investigated the effects of sigh associated with PCV and PSV on the lungs, diaphragm, and distal organs in experimental models of mild ALIp and ALIexp with similar lung mechanical impairment in rats.

## Methods

All procedures were approved by the Ethics Committee of the Health Sciences Center, Federal University of Rio de Janeiro (CEUA 019), and complied with laboratory animal welfare principles.

### Animal preparation and experimental protocol

Forty-eight male Wistar rats (weight 300 to 350 g) were randomly assigned to mild ALI induced by the administration of *Escherichia coli* lipopolysaccharide (LPS), O55:B5) either intratracheally (200 μg) (pulmonary ALI, ALIp group) or intraperitoneally (1,000 μg) (extrapulmonary ALI, ALIexp group), suspended in saline solution to a total volume of 100 μl and 1,000 μl respectively [8,9]. For intratracheal instillation of LPS, rats were first anesthetized with sevoflurane. These doses of *E. coli* LPS have been reported in a previous study [8] to yield a similar 1.5-fold-increase in static lung elastances in ALIp and ALIexp groups compared to controls. Twenty-four hours after ALI induction, the rats were sedated (10 mg/kg diazepam, intraperitoneally), anesthetized (100 mg/kg ketamine and 10 mg/kg xylazine, intraperitoneally), and tracheotomized, and a snugly fitting cannula 1.5 mm in inner diameter and 6.8 mm in length was introduced into the trachea.

**Figure 1:**
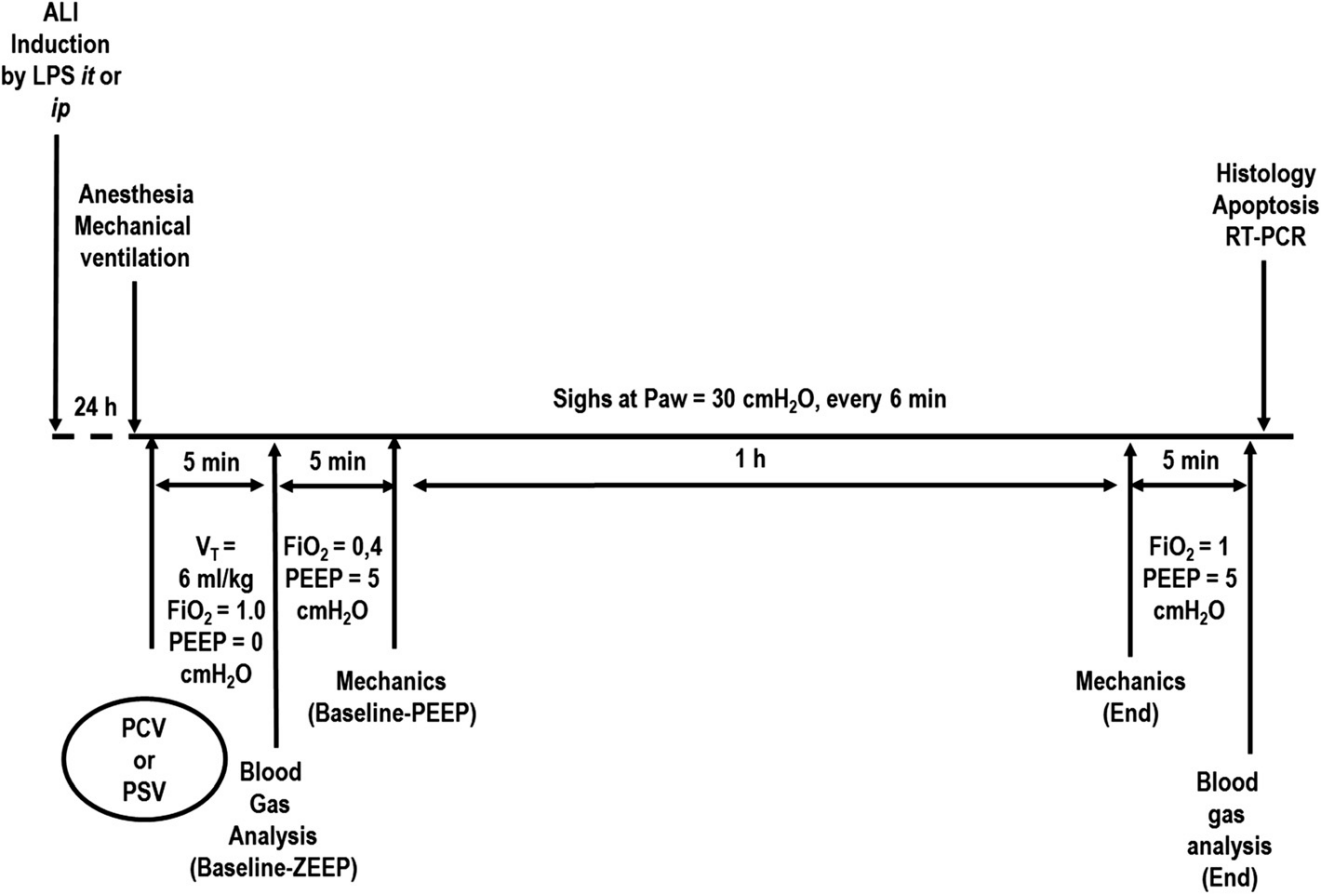
**Timeline representation of the experimental procedure.** ALI, acute lung injury; FiO_2_, fraction of inspired oxygen; i.t., intratracheal; i.p., intraperitoneal; LPS, lipopolysaccharide; Paw, airway pressure; PCV, pressure-controlled ventilation; PEEP, positive end-expiratory pressure; PSV, pressure-support ventilation; RT-PCR, real-time reverse transcription polymerase chain reaction; V_T_, tidal volume; ZEEP, zero end-expiratory pressure.

A polyethylene catheter (PE-10) was introduced into the carotid artery for blood sampling and monitoring of mean arterial pressure (MAP). Electrocardiogram (EKG), MAP and rectal temperature were continuously recorded (Networked Multi-Parameter Veterinary Monitor LifeWindow™ 6000 V, Digicare Animal Health, Boynton Beach, FL, USA). The tail vein was punctured for continuous infusion of Ringer’s lactate (10 ml/kg/h). Gelafundin™ (B. Braun, Melsungen, Germany) was administered (in 0.5 ml increments) to keep MAP >70 mmHg. Animals were mechanically ventilated (Servo-i, MAQUET, Solna, Sweden) in PCV or PSV. During PCV, animals were paralyzed with pancuronium bromide (2 mg/kg, intravenously). In PCV and PSV, the driving pressure was adjusted to achieve V_T_ = 6 ml/kg. In addition, in PCV, the respiratory rate (RR) was controlled to keep minute ventilation constant (160 ml/min). For Baseline-zero end-expiratory pressure (ZEEP), the fraction of inspired oxygen (FiO_2_) was adjusted to 1.0 over 5 min to evaluate the oxygenation impairment induced only by the intratracheal or intraperitoneal administration of LPS. Arterial blood (300 μl) was drawn into a heparinized syringe to determine arterial oxygen partial pressure (PaO_2_), arterial carbon dioxide partial pressure (PaCO_2_), and arterial pH (pHa) (i-STAT, Abbott Laboratories, Abbott Park, IL, USA). For Baseline-PEEP, PEEP was set at 5 cmH_2_O and FiO_2_ = 0.4 and mechanical data obtained. Animals in the PCV or PSV groups were then randomly assigned to the following subgroups: (1) non-Sigh (NS) (n = 6) or (2) 10 sighs/hour (Sigh: manually every 6 min, n = 6) with an inspiratory plateau pressure of 30 cmH_2_O. Each sigh lasted 0.66 seconds, which is double the inspiratory time in relation to a regular cycle in PCV. After 1 h of mechanical ventilation, FiO_2_ was set at 1.0. After 5 minutes, arterial blood gases were analyzed at PEEP 5 cmH_2_O (End). The animals were sacrificed and their lungs extracted for histological and molecular biology analysis (Figure 1).

### Data acquisition and processing

A pneumotachograph (internal diameter = 1.5 mm, length = 4.2 cm, distance between side ports = 2.1 cm) was connected to the tracheal cannula for airflow (V') measurements. The pressure gradient across the pneumotachograph was determined using a SCIREQ differential pressure transducer (UT-PDP-02, SCIREQ, Montreal, QC, Canada). Tidal volume was calculated by digital integration of the flow signal. Airway pressure (P_aw_) was measured with a SCIREQ differential pressure transducer (UT-PDP-300, SCIREQ, Montreal, QT, Canada). Changes in esophageal pressure (P_es_), which reflect chest wall pressure, were measured with a 30-cm-long water-filled catheter (PE205) with side holes at the tip connected to a differential pressure transducer (UT-PL-400, SCIREQ, Montreal, QC, Canada). The catheter was passed into the stomach and then slowly returned into the esophagus; its proper positioning was assessed using the ‘occlusion test’ [11]. Transpulmonary pressure (P,_L_) was calculated during inspiration and expiration as the difference between tracheal and esophageal pressures. Transpulmonary mean pressure (Pmean,_L_), transpulmonary peak pressure (Ppeak,_L_), and the esophageal pressure generated 100 ms after onset of inspiratory effort (P_0.1_) were calculated. The RR was calculated from the P_es_ swings as the frequency per minute of each type of breathing cycle. Airflow and tracheal and esophageal pressures were continuously recorded throughout the experiments with a computer running software written in LabVIEW™ (National Instruments; Austin, TX, USA). All signals were filtered (200 Hz), amplified by a 4-channel conditioner (SC-24, SCIREQ, Montreal, QC, Canada), sampled at 200 Hz with a 12-bit analog-to-digital converter (National Instruments; Austin, TX, USA). All mechanical data were computed offline by a routine written in MATLAB (Version R2007a; The Mathworks Inc, Natik, MA, USA).

### Histology

#### Light microscopy

A laparotomy was performed immediately after blood sampling at the end of experiments. Heparin (1,000 IU) was injected into the tail vein. The trachea was then clamped at end-expiration (PEEP = 5 cmH_2_O) and the abdominal aorta and vena cava were severed, yielding massive hemorrhage and rapid death by exsanguination. The lungs were removed *en bloc* with an end-expiratory pressure of 5 cmH_2_O in all groups to avoid distortion of lung morphometry. The left lung was frozen in liquid nitrogen and immersed in Carnoy’s solution. Lung morphometric analysis was performed using an integrating eyepiece with a coherent system consisting of a grid with 100 points and 50 lines (known length) coupled to a conventional light microscope (Olympus BX51, Olympus Latin America, Rio de Janeiro, Brazil). The volume fractions of the lung occupied by collapsed alveoli, normal pulmonary areas or hyperinflated structures (alveolar ducts, alveolar sacs, or alveoli; maximal chord length in air >120 μm) were determined by the point-counting technique at a magnification of ×200 across 10 random, non-coincident microscopic fields [12].

#### Transmission electron microscopy of the lung and diaphragm

The pathologist or technician working on the electron microscopy images was blinded to the nature of the study. Three slices measuring 2 × 2 × 2 mm were cut from three different segments of the right lung (apex, middle and base of the lung) and diaphragm. They were then fixed in 2.5% glutaraldehyde and phosphate buffer, 0.1 M (pH = 7.4) for electron microscopy analysis (JEOL 1010 Transmission Electron Microscope; Japan Electron Optics Laboratory Co, Tokyo, Japan). For each electron microscopy image (20 per animal), an injury score was determined. The following parameters were analyzed concerning lung parenchyma: damage to alveolar capillary membrane, type II epithelial cell lesion, and endothelial cell damage [10]. The following aspects were assessed on electron microscopy of diaphragm muscle: (1) myofibril abnormalities, defined as disruption of myofibril bundles or disorganized myofibrillar pattern with Z-disk edema, and (2) mitochondrial injury with abnormal swollen mitochondria and abnormal cristae. Pathological findings were graded on a five-point, semi-quantitative, severity-based scoring system as follows: 0 = normal lung parenchyma or diaphragm, 1 = changes in 1 to 25%, 2 = changes in 26 to 50%, 3 = changes in 51 to 75%, and 4 = changes in 76 to 100% of examined tissue.

### Apoptosis assays

To assay cellular apoptosis, terminal deoxynucleotidyl transferase biotin-dUTP nick end labeling (TUNEL) staining was performed by two pathologists unaware of study group allocation. Apoptotic cells were detected using the commercial In Situ Cell Death Detection Kit, Fluorescin (Boehringer, Mannheim, Germany). Nuclei without DNA fragmentation stained blue as a result of counterstaining with hematoxylin. Ten fields per section from regions with apoptotic cells were examined at a magnification of x400. A five-point, semi-quantitative, severity-based scoring system was used to assess apoptosis: 0 = normal lung, liver and kidney; 1 = changes in 1 to 25%; 2 = changes in 26 to 50%; 3 = changes in 51 to 75%; and 4 = changes in 76 to 100% of examined tissue [13].

### Biological markers of inflammation, apoptosis, and fibrogenesis

Quantitative real-time reverse transcription polymerase chain reaction (RT-PCR) was performed to measure biological markers associated with inflammation (interleukin (IL)-1β and IL-6), fibrogenesis (type III procollagen, (PCIII)), and apoptosis (caspase-3). Central slices of the right lung were cut, collected in cryotubes, flash-frozen by immersion in liquid nitrogen, and stored at −80°C. Total RNA was extracted from frozen tissues using the SV total RNA Isolation System (Promega Corporation, Fitchburg, WI, USA), following manufacturer recommendations. RNA concentration was measured by spectrophotometry in a Nanodrop ND-1000 system. First-strand cDNA was synthesized from total RNA using a GoTaq™ 2-STEP RT-qPCR System (Promega Corporation, Fitchburg, WI, USA). Relative mRNA levels were measured with a SYBR green detection system using ABI 7500 real-time PCR (Applied Biosystems, Foster City, CA, USA). PCR primers for target genes were purchased (Invitrogen, Carlsbad, CA, USA). The following primers were used: IL-1β (sense 5′- CTA TGT CTT GCC CGT GGA G −3′, and antisense 5′- CAT CAT CCC ACG AGT CAC A −3′); IL- 6 (sense 5′- CTC CGC AAG AGA CTT CCA G −3′ and antisense 5′- CTC CTC TCC GGA CTT GTG A −3′); PCIII (sense 5′- ACC TGG ACC ACA AGG ACA C −3′ and antisense 5′- TGG ACC CAT TTC ACC TTT C −3′); caspase-3 (sense 5′- GGC CGA CTT CCT GTA TGC −3′ and antisense 5′- GCG CAA AGT GAC TGG ATG −3′); and GAPDH (sense 5′- GGT GAA GGT CGG TGTG AAC- 3′ and antisense 5′- CGT TGA TGG CAA CAA TGT C −3′). Samples were measured in triplicate. For each sample, the expression of each gene was normalized to expression of the housekeeping gene glyceraldehyde-3-phosphate dehydrogenase (GAPDH) using the 2^–ΔΔCt^ method, where ΔCt = Ct, reference gene – Ct, target gene. The relative expression of each gene was calculated as a ratio compared with the reference gene and expressed as fold change relative to animals ventilated with non-Sigh PCV.

### Statistical analysis

Sample size calculation for testing the primary hypothesis (alveolar collapse is reduced after PSV compared to PCV in a model of experimental pulmonary ALI in rats) was based on effect estimates obtained from pilot studies. Accordingly, we expected that a sample size of six animals per group (providing for one animal as dropout) would provide the appropriate power (1-β = 0.8) to identify significant (α = 0.05) differences in alveolar collapse between controlled and spontaneous breathing, taking into account mean difference = 11.5, standard deviation = 6.3, a two-sided test, and sample size ratio = 1. Sample size calculation was performed in OpenEpi 3.01 (Andrew G. Dean and Kevin M. Sullivan, Atlanta, GA, USA).

Normality of data was tested using the Kolmogorov-Smirnov test with Lilliefors’ correction, while the Levene median test was used to evaluate the homogeneity of variances. If both conditions were satisfied, two-way ANOVA followed by Tukey’s test was used. To compare respiratory parameters and arterial blood gases between Baseline and End, the paired *t* test was used. One-way ANOVA on ranks followed by Dunn’s *post hoc* test was employed to evaluate the semiquantitative analysis of electron microscopy and apoptosis. Parametric data are expressed as mean ± standard deviation (SD), while nonparametric data are expressed as median (interquartile range). The significance level was set at 5%. All statistical tests were performed in GraphPad Prism 5.0 (GraphPad Software, San Diego, CA, USA).

## Results

Mean arterial pressure was higher than 70 mmHg throughout the experiments in both ALI groups. No significant differences among groups were observed in the volume of fluids required to keep MAP higher than 70 mmHg. An additional file shows in more detail the temporal evolution of MAP during the experiment (see Additional file 1).

At Baseline-PEEP and End, tidal volume was comparable among all groups, whereas respiratory rate was lower in the PSV-Sigh than in PCV-Sigh group, regardless of ALI etiology. Sigh led to a significant reduction in Ppeak,L independent of ventilator strategy or ALI etiology. The mean tidal volume during sighs was 5.68 ± 0.38 ml regardless of ventilator strategy. At End, in both ALIp and ALIexp groups, transpulmonary pressures were comparable between PSV-Sigh and PCV-Sigh. In ALIp and ALIexp, the introduction of sigh was associated with reduced P_0.1_. In ALIexp, P_0.1_ was lower in PSV-Sigh than PSV-NS (Table 1).

**Table 1:** **Respiratory parameters**

		**ALIp**				**ALIexp**			
		**PCV**		**PSV**		**PCV**		**PSV**	
		**NS**	**Sigh**	**NS**	**Sigh**	**NS**	**Sigh**	**NS**	**Sigh**
V_T_ (ml)	Baseline-PEEP	1.9 ± 0.3	1.9 ± 0.3	2.0 ± 0.2	2.1 ± 0.3	1.9 ± 0.2	2.0 ± 0.2	2.0 ± 0.2	2.1 ± 0.3
	End	2.0 ± 0.3	2.1 ± 0.2	2.0 ± 0.3	2.1 ± 0.4	2.0 ± 0.2	1.9 ± 0.2	2.0 ± 0.2	2.2 ± 0.3
RR (bpm)	Baseline-PEEP	76.3 ± 4.5	76.4 ± 3.7	65.9 ± 10.3	57.6 ± 11.1^**^	75.8 ± 3.4	78.1 ± 2.3	60.8 ± 18.1	59.8 ± 17.2^**^
	End	77.3 ± 3.8	76.5 ± 3.7	63.6 ± 8.8	46.3 ± 11.5^**#^	74.1 ± 4.3	78.1 ± 2.3	67.2 ± 14.9	47.3 ± 18.2^**,#^
Ppeak,L (cmH_2_0)	Baseline-PEEP	13.2 ± 2.1	12.1 ± 0.7	17.1 ± 2.4	16.5 ± 6.5^**^	10.7 ± 1.7	12.6 ± 0.7	16.0 ± 1.2^*^	14.4 ± 3.8
	End	13.2 ± 2.3	9.2 ± 0.7^*,†^	14.8 ± 2.8	11.0 ± 1.0^#†^	12.3 ± 2.7	10.0 ± 1.5^†^	14.8 ± 1.6	12.4 ± 3.3^†^
Pmean,L (cmH_2_0)	Baseline-PEEP	8.1 ± 0.9	7.7 ± 0.5	7.8 ± 0.7	7.9 ± 0.8	7.2 ± 0.7	7.9 ± 0.3	7.1 ± 0.8	7.4 ± 1.3
	End	8.2 ± 1.0	6.6 ± 0.3^*,†^	7.4 ± 0.7	6.2 ± 0.3^#†^	7.6 ± 1.1	6.7 ± 0.4^†^	7.2 ± 0.4	6.5 ± 0.9
P_0.1_	Baseline-PEEP	-	-	4.2 ± 2.7	4.6 ± 2.1	-	-	4.2 ± 1.1	2.2 ± 0.4
	End	-	-	2.9 ± 2.2^†^	1.3 ± 0.9^†^	-	-	3.9 ± 0.2	1.3 ± 0.5^†#^

Values are mean + standard deviation (SD) of six rats in each group. †Significantly different from Baseline-PEEP (*P* <0.05); *significantly different from PCV-NS (*P* <0.05); **significantly different from PCV-Sigh (*P* <0.05); ^#^significantly different from PSV-NS (*P* <0.05). V_T_, tidal volume; RR, respiratory rate; Ppeak,L, transpulmonary peak pressure; Pmean,L, transpulmonary mean pressure; P0.1, driving pressure; PEEP, positive-end expiratory pressure; NS, non-Sigh.

There were no significant differences among the groups in relation to pHa, PaCO_2_, and PaO_2_ at Baseline ZEEP and End. Mechanical ventilator strategy and ALI etiology did not affect PaO_2_, PaCO_2_, or pHa after 1 hour of ventilation (End) (Table 2).

**Table 2:** **Blood gas analysis at Baseline-ZEEP and End**

		**ALIp**				**ALIexp**			
		**PCV**		**PSV**		**PCV**		**PSV**	
		**NS**	**Sigh**	**NS**	**Sigh**	**NS**	**Sigh**	**NS**	**Sigh**
pHa	Baseline-ZEEP	7.26 ± 0.05	7.28 ± 0.07	7.30 ± 0.04	7.28 ± 0.04	7.20 ± 0.13	7.18 ± 0.06	7.21 ± 0.05	7.27 ± 0.05
	End	7.25 ± 0.06	7.32 ± 0.05	7.26 ± 0.04	7.21 ± 0.08	7.25 ± 0.07	7.20 ± 0.09	7.25 ± 0.08	7.21 ± 0.11
PaCO_2_ (mmHg)	Baseline-ZEEP	47.3 ± 17.9	43.4 ± 5.2	45.4 ± 3.7	45.8 ± 4.3	49.2 ± 4.6	46.7 ± 7.9	44.6 ± 10.4	44.7 ± 11.7
	End	38.6 ± 14.2	45.3 ± 5.4	51.7 ± 3.3	58.7 ± 7.3	43.5 ± 5.0	38.2 ± 3.0	50.3 ± 8.9	52.8 ± 9.2
PaO_2_ (mmHg)	Baseline-ZEEP	151.8 ± 54.4	136.3 ± 56.7	143.6 ± 33.3	125.7 ± 19.5	126.0 ± 24.6	154.3 ± 38.3	132.8 ± 12.1	134.6 ± 38.8
	End	386.4 ± 91.5	474.7 ± 114.6	409.5 ± 141.7	430.0 ± 169.7	390.2 ± 158.6	468.8 ± 100	437.7 ± 145.2	424.8 ± 127.5

Values are mean + standard deviation (SD) of six rats in each group. Arterial oxygen partial pressure (PaO_2_, mmHg), arterial carbon dioxide partial pressure (PaCO_2_), and arterial pH (pHa) measured at Baseline-ZEEP (zero end-expiratory pressure) and after 1 hour of mechanical ventilation (End) at fraction of inspired oxygen (FiO_2_) = 1.0 in animals with experimentally induced pulmonary (p) and extrapulmonary (exp) acute lung injury (ALI). ALIp, pulmonary acute lung injury; ALIexp, extrapulmonary acute lung injury; PCV, pressure-controlled ventilation; PSV, pressure support ventilation; NS, non-Sigh.

**Figure 2:**
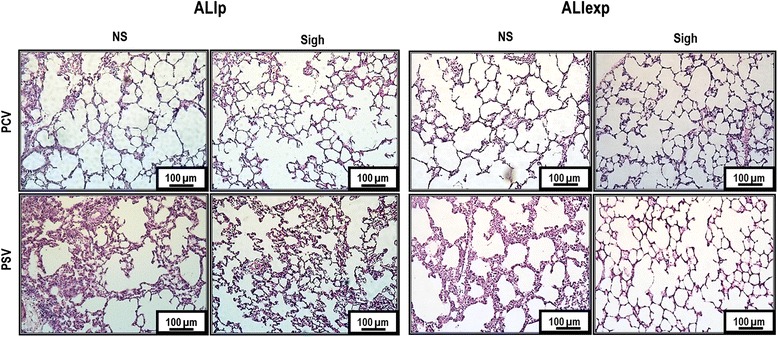
**Photomicrographs of lung parenchyma stained with hematoxylin and eosin.** Photomicrographs are representative of data obtained from lung sections of six animals (original magnification, x200). ALIexp, extrapulmonary acute lung injury; ALIp, pulmonary acute lung injury; PCV, pressure-controlled ventilation; PSV, pressure support ventilation.

**Figure 3:**
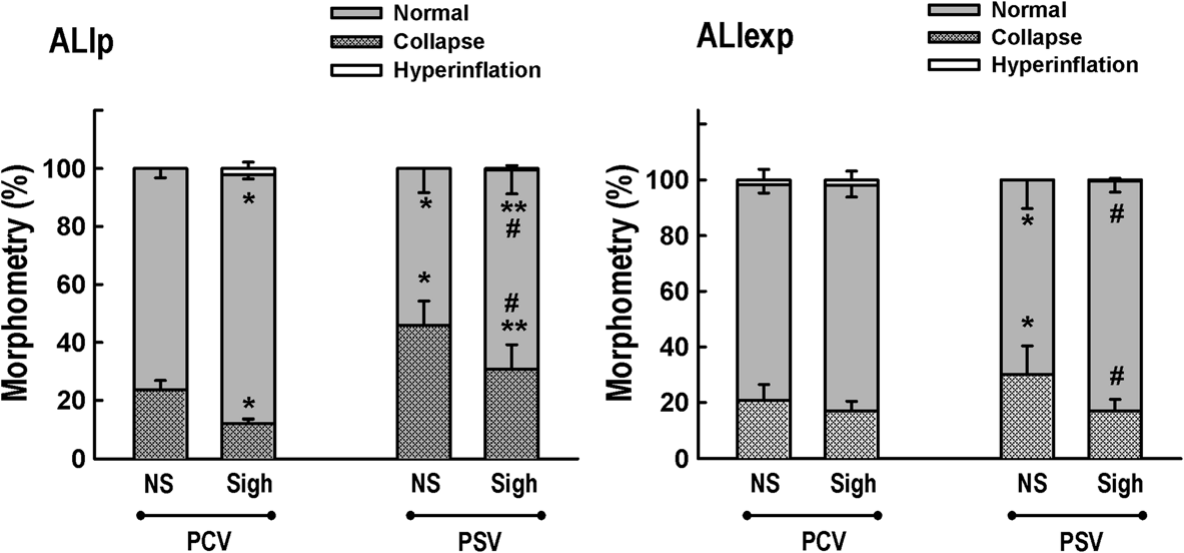
**Volume fraction of the lung occupied by normal pulmonary areas, collapsed alveoli, and hyperinflated structures.** Each bar represents the mean + standard deviation (SD) of six rats in each group. *Significantly different from PCV non-Sigh (*P* <0.05); **significantly different from PCV-Sigh (*P* <0.05); ^#^significantly different from PSV non-Sigh (*P* <0.05). ALIexp, extrapulmonary acute lung injury; ALIp, pulmonary acute lung injury; NS, non-sigh; PCV, pressure-controlled ventilation; PSV, pressure support ventilation.

Figure 2 depicts light microscopy of representative animals of each ventilator strategy and ALI etiology. In ALIp, PCV-Sigh and PSV-Sigh reduced alveolar collapse. In ALIexp, only PSV-Sigh decreased alveolar collapse (Figure 3).

**Figure 4:**
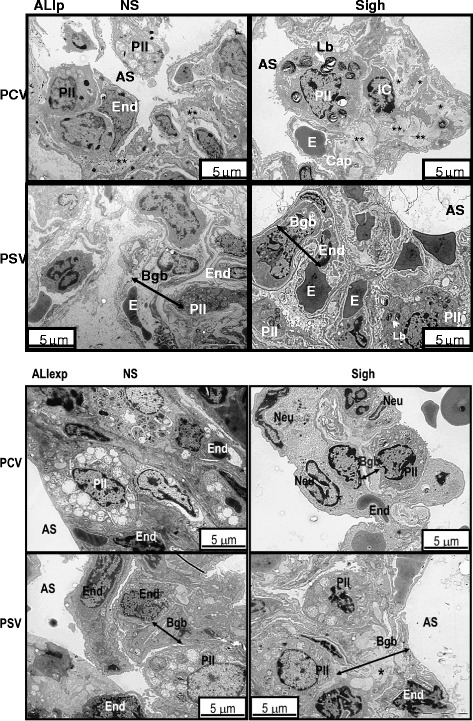
**Electron microscopy of lung parenchyma in ALIp and ALIexp.** Photomicrographs are representative of data obtained from lung sections of five animals per group. Type II epithelial cell (PII) damage with bizarre lamellar bodies (Lb) and apoptosis of epithelial (PII) and endothelial cells (End) are visible in all groups. In ALIp, PCV-Sigh was associated with greater endothelial cell damage as well as with interstitial edema (asterisks), whereas PSV-Sigh was associated with comparatively less alveolar-capillary damage. In ALIexp, the blood-gas barrier (Bgb) was thickened from edema in PSV-Sigh animals compared to PCV-Sigh and PSV-Non-Sigh animals. AS, intra-alveolar space; Cap, capillary; E, erythrocyte; ED, edema; IC, interstitial cell; Neu, neutrophil.

Figure 4 shows electron microscopy findings of lung parenchyma in each group representative animal. Damage to type II epithelial and endothelial cells, as well as alveolar-capillary membrane was independent of ALI etiology (Table 3). In ALIp, PCV-Sigh presented greater damage to alveolar-capillary membrane and endothelial cell compared to PCV-NS. Nevertheless, alveolar-capillary membrane and endothelial cell damage were less pronounced in PSV-Sigh compared to PCV-Sigh. On the other hand, in ALIexp, PSV-Sigh resulted in further damage to the alveolar-capillary membrane, type II epithelial, and endothelial cells (Table 3).

**Table 3:** **Semiquantitative analysis of lung and diaphragm electron microscopy**

**Groups**			**Lung**			**Diaphragm**	
			**Alveolar-capillary membrane**	**Type II epithelial cells**	**Endothelial cells**	**Abnormal myofibril with Z-disk edema**	**Mitochondrial injury**
ALIp	PCV	NS	2 (2-3)	3 (2.75-3)	2 (1.75-2.25)	3 (2.5-3)	2 (2-3)
		Sigh	4 (3-4)*	3 (3-4)	3 (2.75-4)*	2 (2-3)	2 (2-2.5)
	PSV	NS	2 (1.75-2)	2 (2-2.25)*	1 (1-2)	2 (1.5-2)*	2 (1.5-2)
		Sigh	2 (2-3)**	2 (2-2.25)**	2 (2-2)**	2 (1-2)	1(1-2)
ALIexp	PCV	NS	2 (1.75-2.25)	2 (1.75-2.25)	3 (2.75-3)	3 (2-3)	2 (2-3)
		Sigh	2 (2-2.25)	3 (2-3)*	3 (2.75-3.25)	3 (2.5-3)	3 (2-3)
	PSV	NS	2 (1.75-2)	2 (1.75-2)	2 (1.75-2)*	2 (1.5-2)	2(1-2)
		Sigh	3 (3-3.25)**^#^	3 (3-3.25)^#^	3 (3-4)^#^	2 (1.5-2)	2 (1.5-2)

Values expressed as median (interquartile range) of five animals in each group. A five-point, semi-quantitative, severity-based scoring system was used. Pathological findings were graded as: 0 = normal lung parenchyma; 1 = changes in 1 to 25%; 2 = changes in 26 to 50%; 3 = changes in 51 to 75%; and 4 = changes in 76 to 100% of examined tissue. *Significantly different from PCV-NS (*P* <0.05); **significantly different from PCV-Sigh (*P* <0.05): ^#^significantly different from PSV-NS (*P* <0.05). ALIp, pulmonary acute lung injury; PCV, pressure-controlled ventilation; NS, non-sigh; PSV, pressure support ventilation; ALIexp, extrapulmonary acute lung injury.

**Figure 5:**
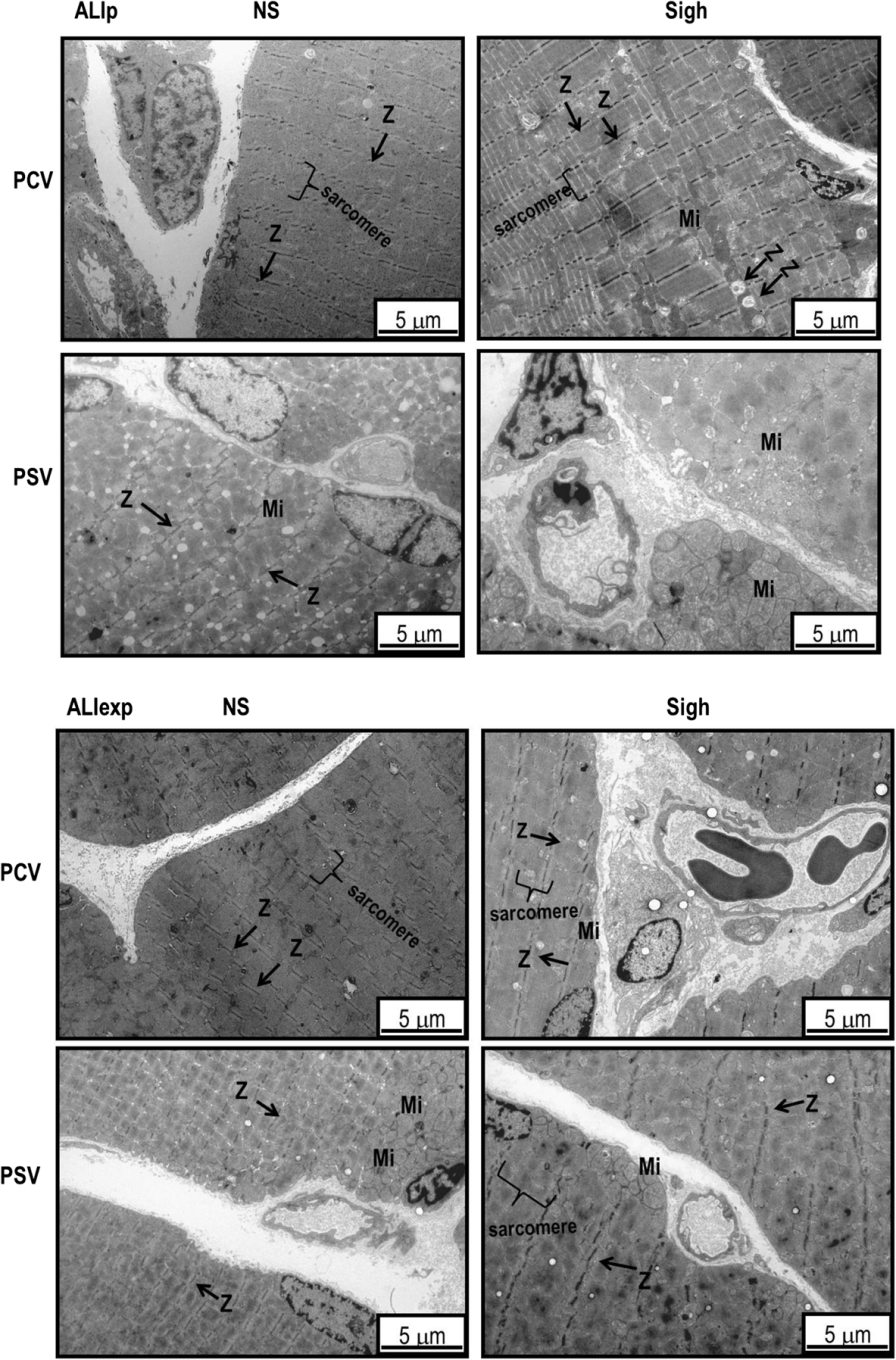
**Electron microscopy of diaphragm specimens in ALIp and ALIexp.** Photomicrographs are representative of data obtained from diaphragm sections of five animals per group. Sigh did not affect the diaphragmatic damage induced by LPS; however, in ALIp, PSV led to less damage than PCV. ALIp, pulmonary acute lung injury; ALIexp, extrapulmonary acute lung injury; LPS, *Escherichia coli* lipopolysaccharide; Mi, mitochondria; PCV, pressure-controlled ventilation; PSV, pressure support ventilation; Z, Z-disk.

Figure 5 depicts electron microscopy findings of diaphragm specimens in each group representative animal. As shown in Table 3, diaphragm damage was greater in PCV-NS than PSV-NS, in ALIp.

In ALIp, no significant changes were observed in the number of apoptotic cells in lung, liver, and kidney between the different ventilator strategies. In ALIexp, the number of apoptotic cells in the lung was higher in PCV-Sigh compared to PCV-NS (Table 4).

**Table 4:** **Cell apoptosis in lung and distal organs**

	**ALIp**				**ALIexp**			
	**PCV**		**PSV**		**PCV**		**PSV**	
	**NS**	**Sigh**	**NS**	**Sigh**	**NS**	**Sigh**	**NS**	**Sigh**
Lung	2 (2-3)	2 (2-3)	2 (1-2)	2 (1.5-2)	1 (1-2)	3 (2-3)*	1 (1-1.25)	2 (2-2.25)
Liver	1 (1-1.5)	1 (1-1.5)	2 (1-2)	1 (1-2)	2 (2-3)	2 (2-3)	2 (2-2.25)	2 (2-3)
Kidney	1 (1-2)	2 (1-2)	1 (1-1.5)	1 (1-2)	3 (2-3)	2 (2-3)	2 (2-2.25)	3 (2-3)

Values expressed as median (interquartile range) of five animals in each group. A five-point, semiquantitative, severity-based scoring system was used. Pathological findings were graded as: 0 = normal lung parenchyma; 1 = changes in 1 to 25%; 2 = changes in 26 to 50%; 3 = changes in 51 to 75%; and 4 = changes in 76 to 100% of examined tissue. *Significantly different from PCV-NS (*P* <0.05). ALIp, pulmonary acute lung injury; ALIexp, extrapulmonary acute lung injury; PCV, pressure-controlled ventilation; PSV, pressure support ventilation; NS, non-sigh.

**Figure 6:**
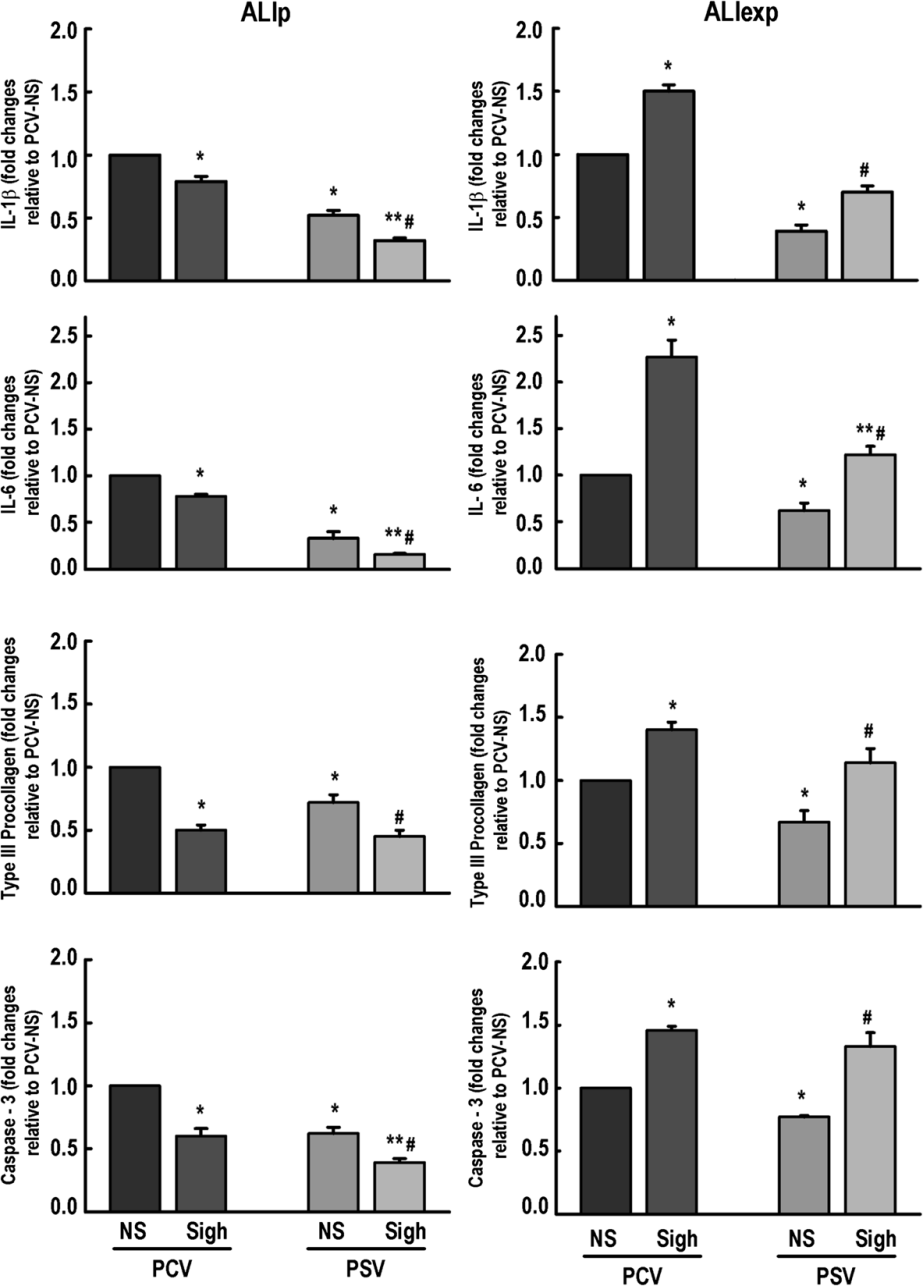
**Expression of biological markers.** Real-time polymerase chain reaction analysis of biological markers associated with inflammation (interleukin (IL)-1β, IL-6), fibrogenesis (type III procollagen), and apoptosis (caspase-3). Relative gene expression was calculated as a ratio of the average gene expression levels compared with the reference gene (GAPDH) and expressed as fold change relative to PCV-NS (non-Sigh). Values are mean + standard deviation (SD) of five rats in each group. *Significantly different from PCV non-Sigh (*P* <0.05); **significantly different from PCV-Sigh (*P* <0.05); ^#^significantly different from PSV non-Sigh (*P* <0.05). ALIp, pulmonary acute lung injury; ALIexp, extrapulmonary acute lung injury; PCV, pressure-controlled ventilation; PSV, pressure support ventilation; NS, non-Sigh.

The mRNA expression of biological markers associated with inflammation, fibrogenesis, and apoptosis is shown in Figure 6. In ALIp, PCV-Sigh and PSV-Sigh showed a reduction in IL-1β, IL-6, PCIII, and caspase-3 mRNA expressions in lung tissue. In ALIexp, PCV-Sigh and PSV-Sigh led to an increase in IL-1β, IL-6, PCIII, and caspase-3 mRNA expressions in lung tissue.

## Discussion

In rat models of mild ALIp and ALIexp tested herein, we found that: (1) PCV-Sigh and PSV-Sigh reduced transpulmonary pressure, and (2) PSV-Sigh decreased the respiratory drive compared to PSV-NS. In ALIp: (1) PCV-Sigh and PSV-Sigh reduced alveolar collapse, IL-1β, IL-6, caspase-3, and PCIII expressions in lung tissue, whereas PCV-Sigh increased alveolar-capillary membrane and endothelial cell damage, and (2) abnormal myofibril with Z-disk edema was greater in PCV-NS than PSV-NS. In ALIexp: (1) PSV-Sigh reduced alveolar collapse, but led to damage to alveolar-capillary membrane, type II epithelial and endothelial cells, (2) PCV-Sigh and PSV-Sigh increased IL-1β, IL-6, caspase-3, and PCIII expressions, and (3) PCV-Sigh increased the number of apoptotic cells in the lung compared to PCV-NS (Table 5). To the best of our knowledge, no previous experimental study has investigated the biological impact of sigh associated with PCV and PSV on lung morphology, inflammation, apoptosis, fibrogenesis, and diaphragm damage in ALIp and ALIexp.

**Table 5:** **Summary of the comparison of sighs in this study**

	**ALIp**				**ALIexp**			
	**PCV**		**PSV**		**PCV**		**PSV**	
	**NS**	**Sigh**	**NS**	**Sigh**	**NS**	**Sigh**	**NS**	**Sigh**
Ppeak,L (cmH_2_0)	→	↓	→	↓	→	↓	→	↓
Pmean,L (cmH_2_0)	→	↓	→	↓	→	↓	→	→
P_0.1_	-	-	→	↓	-	-	→	↓
PaO_2_ (mmHg)	↑	↑	↑	↑	↑	↑	↑	↑
Alveolar collapse	→	↓	→	↓	→	→	→	↓
Overdistension	-	-	-	-	-	-	-	-
Alveolar-capillary membrane injury	→	↑	→	↑	→	→	→	↑
Lung apoptosis	→	→	→	→	↑	→	→	→
mRNA IL-1β	↑	↓	↑	↓	↓	↑	↓	↑
mRNA IL-6	↑	↓	↑	↓	↓	↑	↓	↑
mRNA PCIII	↑	↓	↑	↓	↓	↑	↓	↑
mRNA pro-caspase-3	↑	↓	↑	↓	↓	↑	↓	↑

The arrows indicate the direction of change of each variable relative to respective NS. ↑: increase in relation to NS; ↓: decrease in relation to NS; →: no changes. ALIp, pulmonary acute lung injury; ALIexp, extrapulmonary acute lung injury; PCV, pressure-controlled ventilation; PSV, pressure support ventilation; NS, non-sigh; Ppeak,L, transpulmonary peak pressure; Pmean,L, transpulmonary mean pressure; P0.1, driving pressure; PaO_2_, mmHg, arterial oxygen partial pressure; mRNA IL-1β, mRNA interleukin (IL)-1 β, mRNA IL-6, mRNA interleukin (IL)-6, mRNA PCIII, mRNA type III procollagen.

ALIp and ALIexp were experimentally induced by intratracheal and intraperitoneal injection of *E. coli* LPS respectively [8], yielding similar deterioration of oxygenation (Table 2) and alveolar collapse (Figure 3). In our study, these experimental models led to histological features of ALI [14], such as thickening of the alveolar wall, inflammation, and changes of the alveolar-capillary barrier. ALIp primarily affects the alveolar epithelium, with damage occurring mainly in the intra-alveolar space, with alveolar flooding and areas of consolidation [8]. In ALIexp, endothelial cells are the first target of damage, with a subsequent increase in vascular permeability. Thus, the main pathologic alteration due to an indirect insult may be microvessel congestion and interstitial edema, with relative sparing of intra-alveolar spaces [8]. To minimize the impact of possible confounding factors on distal organ apoptosis, MAP was maintained at 70 mmHg or higher in all animals. The frequency and type of sighs were chosen on the basis of previous studies [15] suggesting that the use of a lower sigh frequency (10 sighs/hour) and limiting plateau pressure to 30 cmH_2_O [16] led to a protective effect on the lung and distal organs in mild experimental ALI. All animals were mechanically ventilated with low V_T_ (6 ml/kg) and the PEEP level was set at 5 cmH_2_O on the basis of previous observations from our group, which suggested that higher levels may result in lung injury in these models of ALI in rats [8,9]. We measured the gene expression of IL-6, IL-1β, PCIII, and caspase-3, because these biomarkers have been associated with inflammation [17], fibrogenesis [18], and apoptosis [19], respectively.

Our results are in line with those reported in previous studies of patients with early ALI, showing that three sighs per minute during controlled mechanical ventilation [4] or one sigh per minute during PSV [6,20] promoted alveolar recruitment. The reduction in atelectatic areas can be explained by progressive recruitment of collapsed alveoli, induced by periodic higher transpulmonary pressures during sigh in both PCV and PSV. The positive effect of sigh on lung morphology was associated with a reduction of Ppeak,L and Pmean,L during conventional controlled or assisted breaths in both ALIp and ALIexp. Additionally, our data show a beneficial interaction between active breathing and sigh. In fact, during PSV, sigh reduced the inspiratory drive measured by P_0.1_ [20], which is consistent with previous studies in patients with ARDS [6,20].

In ALIp, both PCV-Sigh and PSV-Sigh groups presented reduced inflammatory, fibrogenic, and proapoptotic markers whether combined with PCV or PSV (Figure 6, Table 5). This finding may be explained by the opening of consolidated alveoli, thus reducing alveolar collapse and shear stress. However, during PCV, sigh promoted alveolar-capillary membrane and endothelial cell damage. The dissociation between the protective effect on biological markers and the ultrastructural damage to lung parenchyma may be explained by the fact that lung inflation distends type I epithelial cells almost twice as much as type II epithelial cells [21]. As a result, when these cells are submitted to homogeneous ventilation in PSV, in comparison to PCV, even in the absence of sighs, a lower severity score in type II epithelial cells was observed (Table 3). Furthermore, in 1-hour mechanical ventilation abnormal myofibril with Z-disk edema was seen in PCV-NS compared to PSV-NS (Table 3).

There is growing evidence that the endothelial side of the alveolar capillary membrane plays an important role in VILI [22]. In contrast to ALIp, in ALIexp, there was an increased activation of biological markers of inflammation, fibrogenesis, and apoptosis induced by sigh during both PCV and PSV, which might be associated with higher endothelial cell activation and microvessel congestion [23]. In this line, ultrastructural analysis of endothelial cells showed less endothelial ultrastructural damage in PSV-NS compared to PCV-NS (Table 3).

### Possible clinical implications

Changes in lung pressures and oxygenation have limited value in evaluating the effects of sigh associated with PCV or PSV to minimize alveolar collapse and VILI. However, the biological impact of sigh differed according to the etiology of ALI and ventilatory strategy. Our experimental data needs to be confirmed in clinical studies before clinicians consider sigh for the improvement of lung function and protection during PSV in mild lung injury.

### Limitations

This study has several limitations: (1) as ALI models were induced by LPS, care should be taken when attempting to extrapolate our findings either to other ALI models, with different degrees of severity, or to the clinical setting; (2) we cannot rule out possible beneficial effects on VILI induced by the intrinsic variability of breathing pattern during PSV. Nevertheless, the variable patterns might have been reduced due to animal sedation [24] and to the severity of the underlying disease itself [25]; (3) the observation time was relatively short (1 h mechanical ventilation), precluding extrapolation of our findings to longer periods of ventilation. However, prolonging mechanical ventilation to more than 6 h in the current experimental models would also have some limitations: (1) only changes in IL-6 protein levels were observed, since protein synthesis of PCIII and caspase-3 requires more than 6 h, and (2) keeping small animals with ALI alive for 6 h requires administration of larger volumes of fluids, sometimes vasoactive drugs (for example, noradrenaline) to keep MAP higher than 70 mmHg, and bicarbonate to counteract intense metabolic acidosis. All these therapeutic strategies interfere with individual gene activation. Therefore, as a primary study design, even though a 1-h duration represents a short study time, we are able to better evaluate the gene activation induced by the sigh associated with PCV and PSV in different ALI etiologies without the interference of therapies necessary to keep the animals alive; (4) the expression of mediators was quantified using RT-PCR instead of enzyme-linked immunosorbent assay. It is well known that 1 h is sufficient time to produce changes in mRNA expression, but not to change protein levels significantly [10,26,27]; (5) a fixed PEEP level (5cmH_2_O) was used, as it was associated with beneficial effects on lung recruitment in the rat models of ALI used in this study [10,28,29]; and (6) a low tidal volume (6 mL/kg) was used regardless of the mode of ventilation. However, there was a trend toward increase in carbon dioxide during PSV (Table 2). Thus, we cannot rule out that changes in PaCO_2_ may have influenced the inflammatory process [30].

## Conclusions

In the rat models of mild ALI tested in this study, sigh improved lung protection only during PSV in ALIp. This experimental study is the first step to other experimental and clinical studies in order to evaluate the effects of sigh associated with PCV and PSV in pulmonary and extrapulmonary ALI models.

## Key messages

- In PSV, sigh reduced the respiratory drive, regardless of ALI etiology.
- In ALIp, sigh decreased alveolar collapse, with a reduction in inflammation and markers associated with fibrogenesis and apoptosis.
- In ALIexp, sigh reduced alveolar collapse only in PSV, but increased markers associated with inflammation, apoptosis, and fibrogenesis in both PCV and PSV.

## Additional file

Additional file 1: Table S1. Mean arterial pressure.

## Abbreviations

ALI: acute lung injury
ALIexp: extrapulmonary acute lung injury
ALIp: pulmonary acute lung injury
ARDS: acute respiratory distress syndrome
AS: intra-alveolar space
Bgb: blood-gas barrier
Cap: capillary
E: erythrocyte
ED: edema
EKG: electrocardiogram
FiO_2_: fraction of inspired oxygen
GADPH: glyceraldehyde-3-phosphate dehydrogenase
I:E: inspiratory, expiratory ratio
IC: interstitial cell
IL: interleukin
LPS: *Escherichia coli* lipopolysaccharide
MAP: mean arterial pressure
Mi: mitochondria
Neu: neutrophil
NS: non-sigh
P_0.1_: decay in airway pressure 100 ms after start of inspiration
PaCO_2_: partial pressure of arterial carbon dioxide
PaO_2_: partial pressure of arterial oxygen
P_aw_: airway pressure
PCIII: type III procollagen
PCV: pressure-controlled ventilation
PEEP: positive end-expiratory pressure
P_es_: esophageal pressure
pHa: arterial pH
P_L_: transpulmonary pressure
P_peak_: peak airway pressure
P_pl,mean_: mean transpulmonary pressure
PSV: pressure support ventilation
RM: recruitment maneuver
RR: respiratory rate
RT-PCR: real-time reverse transcription polymerase chain reaction
SD: standard deviation
TUNEL: terminal deoxynucleotidyl transferase biotin-dUTP nick end labeling
VILI: ventilator-induced lung injury
V_T_: tidal volume
Z: z-disk
ZEEP: zero end-expiratory pressure
