# Supplementary material for: Effects of sigh during pressure control and pressure support ventilation in pulmonary and extrapulmonary mild acute lung injury
Source: Crit Care. 2014 Aug 12;18(4):474. doi: 10.1186/s13054-014-0474-4 (PMC4155110; doi:10.1186/s13054-014-0474-4)
Supplement: Additional file 1: Table S1. — Mean arterial pressure. [file 13054_2014_474_MOESM1_ESM.pdf]

Additional File 1 **Table S1.** Mean Arterial Pressure

MAP	ALIp				ALIexp			
	PCV		PSV		PCV		PSV	
Time (min)	NS	Sigh	NS	Sigh	NS	Sigh	NS	Sigh
Baseline PEEP	82.8±12.1	92.0±20.6	125.4±18.8	107.4±20.9	102.4±22.9	101.0±20.2	120.3±25.4	121.4±23.0
15	90.1±18.5	85.8±15.5	113.8±15.1	102.1±24.7	106.8±10.1	95.2±16.0	115.5±25.8	116.5±23.4
30	92.1±14.4	89.8±13.7	112.5±14.9	99.6±20.3	110.0±13.9	89.1±14.9	107.3±38.7	109.5±27.8
45	96.3±15.3	94.7±10.3	114.9±11.2	96.7±19.3	107.3±17.5	84.9±22.1	118.2±25.3	107.3±28.2
60	94.9±15.4	101.1±20.4	108.0±13.3	94.0±17.0	110.0±16.6	88.6±22.2	114.3±24.4	110.6±23.5

Mean arterial pressure (MAP) at different time points (Baseline PEEP, 15, 20, 45, and 60 min). Values are mean ± SD of six rats in each group. PCV: pressure control ventilation; PSV: pressure support ventilation; NS: non-sigh.
